# A spatial-temporal continuous dataset of the transpiration to evapotranspiration ratio in China from 1981–2015

**DOI:** 10.1038/s41597-020-00693-x

**Published:** 2020-10-27

**Authors:** Zhongen Niu, Honglin He, Gaofeng Zhu, Xiaoli Ren, Li Zhang, Kun Zhang

**Affiliations:** 1 Key Laboratory of Ecosystem Network Observation and Modeling, Institute of Geographic Sciences and Natural Resources Research, Chinese Academy of Sciences, Beijing, 100101 China; 2 National Ecosystem Science Data Center, Institute of Geographic Sciences and Natural Resources Research, Chinese Academy of Sciences, Beijing, 100101 China; 3 University of Chinese Academy of Sciences, Beijing, 100049 China; 4 College of Resources and Environment, University of Chinese Academy of Sciences, Beijing, 100049 China; 5 Key Laboratory of Western China’s Environmental Systems (Ministry of Education), College of Earth and Environmental Sciences, Lanzhou University, Lanzhou, 730000 China; 6 Institute of Tibetan Plateau Research, Chinese Academy of Sciences, Beijing, 100101 China

**Keywords:** Ecosystem ecology, Hydrology

## Abstract

The ratio of plant transpiration to total terrestrial evapotranspiration (T/ET) captures the role of vegetation in surface-atmosphere interactions. However, several studies have documented a large variability in T/ET. In this paper, we present a new T/ET dataset (also including transpiration, evapotranspiration data) for China from 1981 to 2015 with spatial and temporal resolutions of 0.05° and 8 days, respectively. The T/ET dataset is based on a model-data fusion method that integrates the Priestley-Taylor Jet Propulsion Laboratory (PT-JPL) model with multivariate observational datasets (transpiration and evapotranspiration). The dataset is driven by satellite-based leaf area index (LAI) data from GLASS and GLOBMAP, and climate data from the Chinese Ecosystem Research Network (CERN). Observational annual T/ET were used to validate the model, with *R*^2^ and RMSE values were 0.73 and 0.07 (12.41%), respectively. The dataset provides significant insight into T/ET and its changes over the Chinese terrestrial ecosystem and will be beneficial for understanding the hydrological cycle and energy budgets between the land and the atmosphere.

## Abstract (summary)

**Table:** 

Measurement(s)	ratio of transpiration to total terrestrial evapotranspiration • evapotranspiration • transpiration
Technology Type(s)	model-data fusion • computational modeling technique • digital curation
Factor Type(s)	year of data collection
Sample Characteristic - Environment	terrestrial biome
Sample Characteristic - Location	China

Machine-accessible metadata file describing the reported data: 10.6084/m9.figshare.12917642

## Background & Summary

Evapotranspiration (ET) is a keystone climate variable that uniquely links the hydrological cycle, energy budget, and carbon cycle^1,2^. This process consists of physical evaporation (soil evaporation and canopy interception evaporation) and biological transpiration (T)^3^. Quantifying the ratio of transpiration to total evapotranspiration (T/ET) is an important topic of research^4^, it is crucial for estimating the land water flux and providing insight into the interactions between the terrestrial ecosystem and atmosphere^5,6^. Furthermore, long-term time series of spatially and temporally continuous T/ET products can be used to generate relatively more accurate carbon cycle projections because the biological process impacts of transpiration also control carbon dioxide exchange between the land and atmosphere^7^. This can help improve our understanding of feedback mechanisms between environmental factors and hydrological components, especially within the context of climate change^8,9^.

Multiple approaches have been developed to estimate T/ET at global or regional scales in recent decades. However, their values are still subject to debate^1,10–16^. For example, one isotope-based method indicated that T/ET was approximately 0.80–0.90 at the global scale^1^, which may be an overestimation^17,18^. Another study reported global T/ET values of approximately 0.64 ± 0.13 using the same method^11^. Moreover, the isotope-based method is restricted by the observation period; therefore, it is difficult to obtain long-term data series. Models provide an effective way of estimating T/ET on both temporal and spatial scales. However, due to the inaccurate representation of canopy light use, interception loss and root water uptake process in earth system models, the Coupled Model Intercomparison Project phase 5 (CMIP5) models underestimate T/ET, with a mean value of 0.41 ± 0.11^7^. Additionally, these models cannot capture the opposing trends of transpiration and soil evaporation under terrestrial ecosystem greening^19^, which has been widely monitored around the world, especially in China^9^. The large T/ET variability reported by previous studies suggests that accuracy continues to be a challenge^20^. Moreover, to the best of our knowledge, no spatial-temporal continuous and open access T/ET product exists for the entire Chinese terrestrial ecosystem.

Site measurements can provide accurate local information related to T/ET; however, their relative scarcity and inconsistent measurement periods hinder large-scale upscaling^7^. Fortunately, model-data fusion (MDF) methods are available to assist with data analysis and generate links to models^21^. By combining multisite observations with a model, MDF can be used to optimize the generic nature of model parameters within plant functional types. If a model with optimized parameters is proved to perform well in T/ET simulations according to the site measurements within a plant functional type, one can assume that the model will accurately capture the T/ET spatial variations of the plant function type under present or future climate conditions^7,22^. MDF methods are routinely used to optimize model parameters based on evapotranspiration observations alone^23–25^. These studies significantly improved the accuracy of evapotranspiration simulations but still involved large uncertainties in evapotranspiration partitioning^26,27^. To improve the accuracy of evapotranspiration partitioning, Niu *et al*. (2019) applied a model-fusion approach that integrates PT-JPL model with multivariate observation datasets to the estimated T/ET value of the Chinese terrestrial ecosystem.

**Fig. 1:**
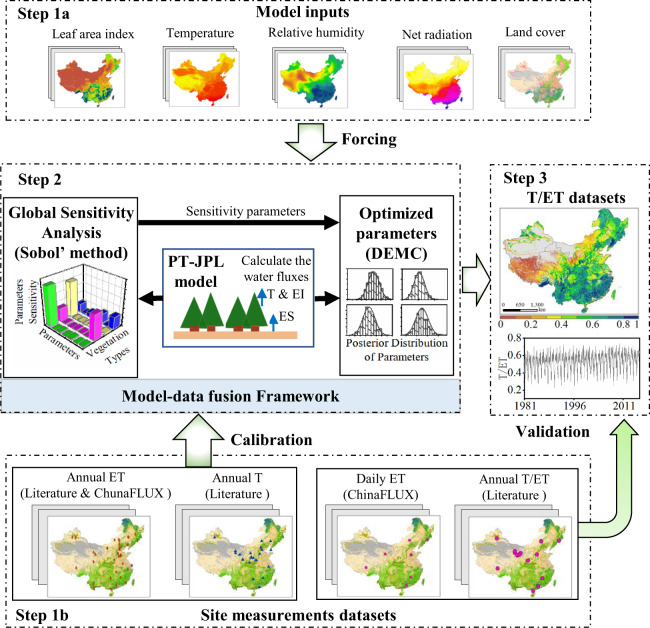
Procedure for producing a spatial-temporal continuous T/ET dataset based on the model-data fusion method. First, model forcing data and constraining data were prepared, respectively. Second, the sensitivity parameters of the PT-JPL model were optimized using the model-data fusion method. Third, the T/ET dataset is calculated using the PT-JPL model with optimization parameters. DEMC stands for Differential Evolution Markov Chain.

The objective of this study is to provide a long-term spatially and temporally consistent T/ET data source with spatiotemporal resolution (0.05°, 8-days and annual) and describe the accuracies of the dataset and method used. The procedure for producing and validating this dataset is shown schematically in Fig. 1 and described in detail in the Methods section. The proposed dataset will be valuable for addressing scientific questions associated with land-atmosphere interactions, global change, and ecological evolution, among others.

## Methods

Three main steps are followed to create the T/ET dataset of Chinese terrestrial ecosystems from 1981–2015:

1. **Data preparation**. An ensemble dataset with 0.05° spatial resolution and 8-day temporal resolution (including leaf area index, net radiation, air temperature, relative humidity) and land cover data were prepared to facilitate the PT-JPL model. Meanwhile, 127 records of annual evapotranspiration for 39 sites, and 48 records of annual transpiration for 26 sites which were obtained from published literature and ChinaFLUX were used to calibrate the PT-JPL model. Annual T/ET for 18 sites collected from published literature and daily evapotranspiration acquired from 6 ChinaFLUX sites were used to validate the PT-JPL model with optimized parameters.
2. **Implementation of MDF method**. We optimized the sensitivity PT-JPL parameters using a model-data fusion method. Firstly, the Sobol’ method was used to identify the sensitivity parameters of the PT-JPL model. Then, the Differential Evolution Markov Chain (DEMC) that integrated the PT-JPL model with multiple observational data (i.e., annual evapotranspiration and transpiration data) was used to optimize the sensitivity parameters.
3. **T/ET simulation**. The PT-JPL model with optimized parameters was used to simulate evapotranspiration and transpiration at the annual and daily scale, respectively. T/ET values were calculated as the quotient of simulated transpiration and evapotranspiration.

Each step is explained in more detail below.

### Data Sets

#### Forcing datasets

We used multiple datasets as the input of the PT-JPL model. All datasets used are summarized in Table 1, and detailed processing methods are described below.

**Table 1:** Input datasets for the Priestley-Taylor Jet Propulsion Laboratory (PT-JPL) model.

Dataset	Model input	Spatial resolution	Temporal resolution	Reference URL
GLASS	Leaf area index	0.05°	8-day	http://www.glass.umd.edu/index.html
GLOBMAP	Leaf area index	0.08°	Half-monthly and 8-day	http://www.modis.cn/
CERN	Meteorological inputs	1 km	8-day	http://www.cnern.org.cn/index.jsp
NESSD	Land cover	1 km	Annual	http://www.geodata.cn/

Leaf area index. LAI is a major input data for the PT-JPL model. Specifically, the arithmetic mean value of Long-term Global Mapping (GLOBMAP) LAI^28^ and Global Land Surface Satellite (GLASS) LAI^29,30^ were used to drive the model. To derive long-term LAI records back to 1981, the GLOBMAP LAI was generated by the quantitative fusion of MODIS and AVHRR data, with a spatial resolution of 0.08°, a half-monthly temporal resolution for the period ranging from 1981 to 2000, and an 8-day temporal resolution for the period ranging from 2001 to 2015. Compared with field measurement data, the GLOBALMAOP LAI has an error of 0.81 LAI on average, with *R*^2^ value being 0.71^28^. The GLASS LAI was also constructed using AVHRR and MODIS data with a general regression neural network, the spatial and temporal resolution was 0.05° and 8 days, respectively. The GLASS LAI values were closer to the mean values of the high-resolution LAI maps, with the *R*^2^ and RMSE values being 0.81 and 0.78, respectively^30^. We first resampled the LAI datasets to 8-day temporal resolution and 0.05° spatial resolution, respectively. Then, the arithmetic mean values of the LAI datasets were calculated as the input of the PT-JPL model.

Meteorological inputs. Meteorological forcing data such as net radiation, mean air temperature, and relative humidity were prepared using the meteorological raster dataset from the Chinese Ecosystem Research Network (CERN). The mean air temperature and relative humidity were generated from 1098 ground meteorological stations using the ANUSPLIN interpolation computer software^31^. The interpolated air temperature data was satisfactory with the *R*^2^ value being 0.94, compared to seven flux monitoring towers in the Asian region^31^. In this study, we compared the interpolated relative humidity with sites measurement data from 6 ChinaFLUX sites (Supplementary Table S1), whereby *R*^2^ and RMSE values were 0.85 and 0.86 (12.72% compared to the average relative humidity), respectively (Supplementary Fig. S1). The net radiation data were calculated from the FAO Penman model based on site measurement data from the China Meteorological Data Service Center, including 53 radiation sites, which were used to optimize parameters, and 699 weather sites, which were used to calculate the net radiation according to the optimized FAO Penman model and measurement data of these stations. Finally, the grid net radiation data were interpolated using ANUSPLIN software^32,33^. Compared with the data from observed sites, the simulated net radiation had a relatively high simulation accuracy with the *R*^2^ value being 0.72, and the mean relative error being 13%^32^. The original data had a temporal and spatial resolution of 8 days and 1 km, and they were resampled to a spatial resolution of 0.05° to match that of the LAI data.

Land cover. Land cover data of China obtained from the National Earth System Science Data Center, National Science & Technology Infrastructure of China, was constructed based on Landsat and GF-2. The classification and overall accuracies were ultimately evaluated through the confusion matrix. The comprehensive evaluation accuracy of the first level of land use was >93% and that of the second level was >90%^34,35^. The vegetation was divided into four types: forest, shrubland, grassland, and cropland. The original spatial resolution was 1 km, which was resampled to 0.05° to match that of LAI data.

#### Calibration and validation datasets

Site measured annual evapotranspiration and transpiration were used to determine the PT-JPL model, and literature collection annual T/ET data and daily evapotranspiration from ChinaFLUX were used to validate the model across different ecosystems (Table 2). The measured sites have a broad spatial distribution and cover all major ecosystem types in China (Supplementary Fig. S2).

**Table 2:** Site measurement data used to calculate and validate the PT-JPL model.

Type	Calibration/Validation	Number of sites				
		Forest	shrubland	grassland	Cropland	ALL
Annual T	Calibration	20	2	3	1	26
Annual ET	Calibration	18	3	8	10	39
Daily ET	Validation	3	1	1	1	6
Annual T/ET	Validation	7	2	6	3	18

Annual evapotranspiration observations. By synthesizing eddy-covariance water flux data in China from both ChinaFLUX observations and published literature, we constructed the dataset of the actual annual evapotranspiration of typical terrestrial ecosystems across China. The dataset contained 127 records of actual annual evapotranspiration for 39 ecosystems, covering 18 forests, 3 shrublands, 8 grasslands, and 10 croplands (Supplementary Table S2)^32,36–59^. The following methods were adopted in the screening of these data: (1) evapotranspiration data were uniformly measured by the eddy-covariance method, (2) only sites with at least one year of continuous flux measurements were included, (3) only one site was selected when there are multiple sites with in a remote-sensing pixels.

Daily evapotranspiration observations. We obtained the complete time series of 30 min evapotranspiration data from 6 sites, covering 3 forests, 1 shrubland, 1 grassland, and 1 cropland. Supplementary Table S1 shows information on these sites. The routine processing procedures recommend by ChinaFLUX were applied to process the evapotranspiration data, including coordinate rotation, Webb-Pearman-Leuning (WPL) correction, storage term calculation, outlier filter, and gap filling^60^. Finally, the 30 min evapotranspiration data were collected to obtain 8-day evapotranspiration data for a given ecosystem.

Annual transpiration observations. Transpiration data were collected from published literature, representing 20 forest, 2 shrub, 3 cropland, and 1 grassland sites (Supplementary Table S3)^61–86^. The following steps were used to screen these data. First, only transpiration data measured by the sap flow method were selected. However, the sap flow method was not applicable for the grassland site; therefore, the transpiration data obtained from the Hydrus-1D model and site measurement data was used in this study. Second, only sites with at least one year of continuous flux measurements were included.

Annual T/ET observations. Field observations of T/ET across 18 sites, including 7 forest, 6 grassland, 2 shrubland, and 3 cropland sites (Supplementary Table S4)^51,85,87–94^ were collected from previous research studies. These studies experimentally measured at least three out of the four relevant variables, that is, evapotranspiration, transpiration, soil evaporation, and interception loss. We only retained site observations that were complete for at least a year or growing season.

### PT-JPL model description

The Priestley-Taylor (PT) equation^95^ is a simplified but successful model for estimating the potential evapotranspiration from a wet surface^95^. In the PT-JPL model, the total evapotranspiration is partitioned into canopy transpiration (*T*), interception evaporation (*EI*), and soil evaporation (*ES*), which are expressed as follows:

1 $$ET=ES+EI+T$$

2 $$ES=({f}_{wet}+{f}_{sm}(1-{f}_{wet})){\rm{\alpha }}\frac{\Delta }{\Delta +\gamma }({R}_{ns}-G)$$

3 $$EI={f}_{wet}{\rm{\alpha }}\frac{\Delta }{\Delta +\gamma }{R}_{nc}$$

4 $$T=(1-{f}_{wet}){f}_{g}{f}_{T}{f}_{M}{\rm{\alpha }}\frac{\Delta }{\Delta +\gamma }{R}_{nc}$$

where *α* is the PT coefficient of 1.26 for a water body (unitless); *Δ* is the slope of the saturation-to-vapor pressure curve (kPa °C^−1^); *γ* is the psychrometric constant (0.066 kPa °C^−1^); *G* is the ground heat flux (W m^−2^); *R*_nc_ is the net radiation to the canopy (W m^−2^), defined as *R*_nc_ = *R*_n_ − *R*_ns_, where *R*_n_ is the net radiation (W m^−2^); and *R*_ns_ is the net radiation to the soil (W m^−2^). *R*_ns_ can be calculated as ${R}_{ns}={R}_{n}{e}^{-{k}_{Rn}LAI}$, where *k*_*Rn*_ is the extinction coefficient (unitless).

PT-JPL effectively accomplishes its partitioning using a canopy extinction equation to estimate the radiation penetrating through the canopy^96^. This canopy extinction equation partition net radiation between the canopy and soil through utilizing the LAI in conjunction with the Beer-Lambert Law of light attenuation. Canopy transpiration are determined by the radiation intercepted according to the Beer-Lambert equation, and transpiration is constrained using four physiological parameters (i.e., *f*_wet_, *f*_g_,*f*_T_, and *f*_M_). Soil evaporation is determined using the residual radiation penetrating the canopy, and that are constrained by surface wetness parameter (*f*_wet_) and the available soil moisture (*f*_sm_). The restricted parameters are described in Table 3.

**Table 3:** Parameters and equations of the PT-JPL model^8,97^.

Parameter	Description	Equation
*f*_wet_	Relative surface wetness	*f*_wet_ = *RH*^4^
*f*_g_	Green canopy fraction	*f*_g_ = *f*_APAR_/*f*_IPAR_
*f*_T_	Plant temperature constraint	${f}_{T}=\exp \left[-{\left(\frac{\left({T}_{a}-{T}_{opt}\right)}{{T}_{opt}}\right)}^{2}\right]$
*f*_SM_	Soil moisture constraint	*f*_SM_ = *RH*^VPD/β^
*f*_M_	Plant moisture constraint	*f*_M_ = *f*_APAR_/*f*_APARMAX_
*f*_APAR_	Fraction of PAR absorbed by the canopy	${f}_{APAR}={b}_{1}(1-{e}^{-{k}_{1}\times LAI})$
*f*_IPAR_	Fraction of PAR intercepted by the canopy	${f}_{IPAR}={b}_{2}(1-{e}^{-{k}_{2}\times LAI})$

RH = relative humidity (%); VPD = saturation vapor pressure deficit (kPa); Ta = air temperature (°C); *T*_opt = _optimum temperature for plant growth (°C); β* = *sensitivity of the soil moisture constraint to VPD (kPa); *f*_APARmax_ = maximum *f*_APAR_; *b*_1_, *b*_2_, *k*_1_, and *k*_2_ = parameters (unitless). Seven parameters need to be estimated: *b*_1_, *b*_2_, *k*_1_, *k*_2,_ *T*_opt_, *β*, and *k*_*Rn*_^8^.

### Model-data fusion method

#### Global sensitivity analysis

The Sobol’ method^98,99^, a globally popular sensitivity analysis technique based on variance decomposition, was integrated with multiple observational data (transpiration and evapotranspiration) to determine the sensitivity of parameters in the PT-JPL model. The Sobol’ method can quantify the sensitivity indices of each parameter based on the partial variance and total variances:

5 $${\rm{First}} \mbox{-} {\rm{order}}\,{\rm{index}}\,{S}_{m}=\frac{{V}_{m}}{V}$$

6 $${\rm{Second}} \mbox{-} {\rm{order}}\,{\rm{index}}\,{S}_{mn}=\frac{{V}_{mn}}{V}$$

7 $${\rm{Total}} \mbox{-} {\rm{order}}\,{\rm{index}}\,{S}_{Tm}={S}_{m}+\sum _{n\ne m}\,{S}_{mn}+\ldots =1-\frac{{V}_{ \sim m}}{V}$$

where *V*_*m*_ is the partial variance with a first-order index of *ϑ*_*m*_ on the model output, *V*_*mn*_ is the partial variance with a second-order index of the *m*th and *n*th parameter interactions, and *V* is the total model variance. *S*_*m*_ is a measure ratio from the main effect of the individual parameter *ϑ*_*m*_ to the total model variance *V*, *S*_*mn*_ defines the sensitivity that results from the interactions between *ϑ*_*m*_ and *ϑ*_*n*_, and *S*_*Tm*_ represents the main effects of *ϑ*_*m*_ and its interactions with the other parameters and can be calculated using the variance *V*_*~m*_, which is the variation of all parameters except *ϑ*_*m*_^25^. Niu *et al*. (2019) offer a detailed description of the implemented computational process.

#### Parameter optimization

To acquire more accurate model simulation results and reduce uncertainties, the DEMC based on Bayes theorem was used to optimize the selected sensitive parameters based on multi-source observed data. This method has been successful at reducing the prior uncertainties of sensitive parameters and improving the accuracy of the model across different biomes^25^. The likelihood function for multivariate data sets *p(O|ϑ)* used for parameter estimation is expressed as the product of the individual *p(O*_*i*_*(∙)|ϑ)*:

8 $$p(O|\theta )=\mathop{\prod }\limits_{i=1}^{I}p({O}_{i}(\cdot )|\theta )$$

9 $$p({O}_{i}(\cdot ){\rm{| }}\theta )=\mathop{\prod }\limits_{t=1}^{{T}_{i}}\frac{1}{\sqrt{2\pi }{\sigma }_{i}}{e}^{-\frac{{({\Delta }_{i}(t))}^{2}}{2{\sigma }_{i}^{2}}}$$

10 $${\sigma }_{i}=\sqrt{\frac{1}{{T}_{i}}\mathop{\sum }\limits_{t=1}^{{T}_{i}}{({\Delta }_{i}(t))}^{2}}s$$

where *I* is the number of datasets, *T*_*i*_ is the total length of observations of the *i*th data set, ${\Delta }_{i}(t)$ is the model-data mismatch, and ${\sigma }_{i}$ (*i* = 1,2) is the standard deviation of the model error of the *i*th dataset^100^. The detailed disequilibrium method is found in the study by Niu *et al*. (2019). Table 4 shows the optimized parameters for each biome.

**Table 4:** Look-up table of key model parameters for different ecosystem types in China^8^.

Ecosystem type	*k*_1_	*k*_2_	*T*_opt_	*β*
Forest	0.57	0.81	Temperature when ${\rm{\max }}\{LAI\times TAVG\times {f}_{apar}\}$	1.28
Shrub	0.56	0.91		1.17
Crop	0.59	0.84		1.43
Grassland	0.59	0.80		0.80

### Simulation of T/ET dataset

The PT-JPL model with optimized parameters was used to directly simulate daily transpiration and evapotranspiration at each grid. For an 8-day temporal scale, T/ET values were calculated as the quotient of simulated transpiration and evapotranspiration. Annual T/ET values were calculated from the annual cumulative transpiration and evapotranspiration. All data within the span of a year were used to calculate the annual cumulative transpiration and evapotranspiration, and data gaps (i.e., the pixels without data) were set as zero.

## Data Records

The dataset includes not only the T/ET, but also the transpiration and evapotranspiration data, which are directly used to calculate T/ET. Two temporal resolutions (daily and annual) are available, with a spatial resolution of 0.05° × 0.05° (Online-only Table 1). T/ET values are unitless for both annual and daily scales, whereas transpiration and evapotranspiration feature units of mm m^−2^ day^−1^ and mm^−2^ m^−2^ a^−1^ for daily and annual scales, respectively. For the annual and daily scale, T/ET values ranged from 0 to 1, and transpiration and evapotranspiration values were greater than zero. Considerable changes among the evapotranspiration components occur during the year, vegetation transpiration was nearly zero due to the cold temperature and low leaf area in northern China in winter. Therefore, T/ET values were also nearly zero or had no value within the same temporal and spatial range. Those pixels were expressed as −9999. Similarly, pixels of desert and construction land were also expressed as −9999.

All data were stored in the Network Common Data Form (NetCDF) files. Data files with different temporal resolution were stored in separate directories, i.e., Annual and Daily (Online-only Table 1). The naming convention for each type of data file was similar. For the annual scale, the naming convention is in accordance with the template Annual_VVVV.nc (e.g. Annual T_ET.nc), where VVVV represents the variable name. Each file contains 35 layers, which represent the annual variable for the period ranging from 1981 to 2015. For the daily scale, the naming convention follows the template Daily_VVVV_TTTT.nc (e.g. Daily_T_ET_1981.nc), where VVVV and TTTT represent the variable name and four-digit year. Each file contains 46 layers, and each layer represents the 8-day average evapotranspiration, transpiration, or T/ET. We stated the starting and ending time of each averaging period in the daily NetCDF files. All datasets^101^ are accessible on Open Science Framework (https://doi.org/10.17605/OSF.IO/MERZN).

**Fig. 2:**
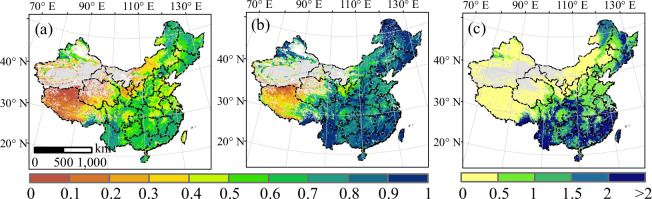
Spatial pattern of annual T/ET, maximum daily T/ET, and annual LAI values in mainland China for 1981–2015. (**a**) Average annual T/ET values, (**b**) maximum daily T/ET values, and (**c**) average annual LAI values (m^2^ m^−2^). The annual T/ET values were obtained from the study by Niu *et al*., (2019).

This study provides a continuous spatial-temporal T/ET dataset. T/ET values for the Chinese terrestrial ecosystem for 1981–2015 range from 0.52–0.59 with a mean value of 0.56. The highest annual T/ET value typically occurs in the east monsoon area, whereas values in the temperate-continental and high-cold Tibetan Plateau areas are relatively low (Fig. 2a). The maximum daily T/ET show similar spatial patterns, but the magnitude is higher than that of the annual mean T/ET values (Fig. 2b). Both the annual T/ET and maximum daily T/ET show spatial patterns that are similar to the leaf area index (Fig. 2c).

## Technical Validation

### Validation using in-site measurements

The accuracy of the simulated T/ET dataset depends on the accuracy of the estimated transpiration and evapotranspiration data^8^. Therefore, the transpiration and evapotranspiration data were first calculated using annual *in-situ* measurements of the Chinese terrestrial ecosystem (Online-only Table 1 and supplementary Fig. S3). With respect to evapotranspiration, upon comparing all sites, forest sites, and non-forest sites with observed data, the *R*^2^ values were 0.71, 0.68, and 0.64, respectively, and the RMSE values were 153.57 (26.21% compared to the multiyear average value, and similarly hereinafter), 147.27 (25.14%), and 116.12 (19.83%), respectively. Meanwhile, the *R*^2^ and RMSE values of regressions between simulated transpiration and observed transpiration were 0.63 and 68.12 (36.80%), respectively. Additionally, the *R*^2^ values were 0.65 and 0.69 for forest sites and non-forest sites, respectively. A complete list of sites used to calibrate evapotranspiration and transpiration are presented in Supplementary Tables S2 and S3, respectively. Compared with other studies, Niu *et al*. (2019) found that the PT-JPL model with optimized parameters was comparable to the performance achieved using models driven by climatic and remote sensing data.

**Fig. 3:**
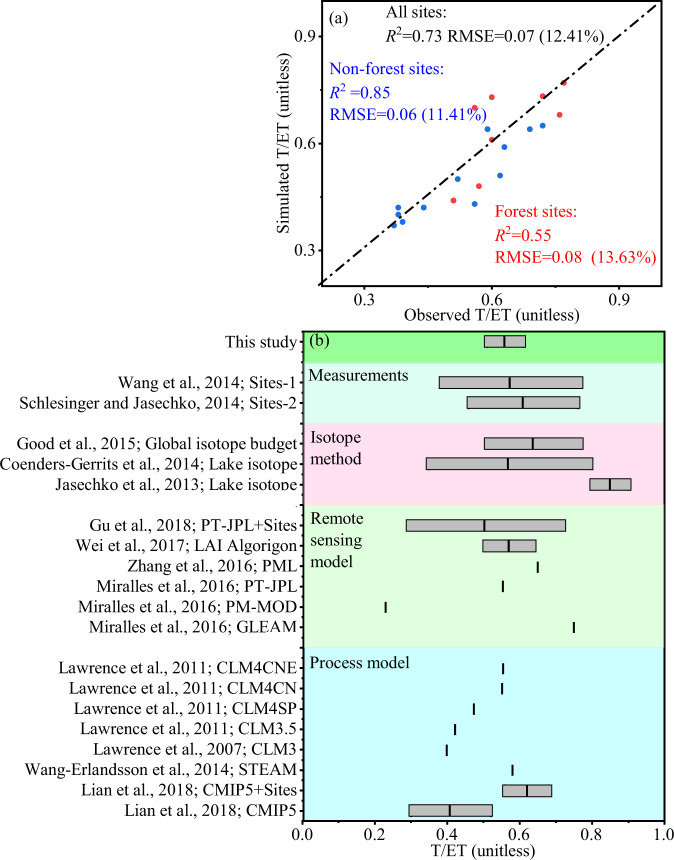
Comparison of simulated T/ET values with those derived from other studies simulated by different methods^1,7,10,11,13,19–21,115–119^.

To further validate the model performance, we compared the simulated annual T/ET to the available filed observation T/ET data in China. A complete list of sites used, and T/ET comparison is presented in Supplementary Table S4. The overall accuracy of the T/ET dataset is relatively high with an *R*^2^ of 0.73 and a low RMSE of 0.07 (12.41%). The T/ET dataset also showed relatively high *R*^2^ and low RMSE for forest and non-forest sites (Online-only Table 1 and Fig. 3a).

We attempted to validate the seasonal dynamic of the simulated database. However, as we were limited by the observational data, only the seasonal fluctuations of evapotranspiration were validated. The *R*^2^ and RMSE values for all sites were 0.75 and 0.62 (35.29%), respectively (Online-only Table 1 and Supplementary Fig. S4). Both the simulated and observed evapotranspiration demonstrated distance seasonal cycles and matched well at different ecosystems (Supplementary Fig. S5). The ET values were high during the plant growing season, and almost zero in the winter due to the limitation of low temperature and plant growth. For the individual ecosystem, the *R*^2^ were all statistically significant at *P* < 0.01 and varied from 0.66 at the evergreen broadleaf forest to 0.91 at the mixed forest, and the RMSE values varied from 26.39% at mixed forests to 44.25% at grasslands.

### Comparison of T/ET magnitude with previous studies

We compared the simulated T/ET magnitude with that reported by previous studies (Fig. 3). Simulated annual T/ET values (0.56 ± 0.05) were within the approximate range of those calculated by process- and remote sensing-based models (0.51 ± 0.08 and 0.56 ± 0.19, respectively). However, the T/ET value was lower than that obtained using the isotope method and from site measurements. The isotope method is constrained by hydrologic decoupling and may have overestimated T/ET^10^. The average T/ET derived from site measurements was 0.60, which was slightly higher than that obtained by this study. Canopy interception evaporation was not included in the majority of data; hence, relatively higher T/ET values were obtained when soil evaporation and transpiration were observed separately^20^.

For Chinese terrestrial ecosystems, the T/ET values simulated by the dataset were close to those derived using measurement data with the S-W model^102,103^. Specifically, the T/ET values estimated for temperate mixed, evergreen coniferous, and evergreen broadleaf forests (0.65, 0.67, and 0.66, respectively) were similar to those reported by Zhu *et al*. (2015) for the same forest types (0.66, 0.67, and 0.67, respectively). Hu *et al*.^102^ reported a range of the annual ratio of evaporation to evapotranspiration of 0.51–0.67 across four grassland ecosystems. Therefore, T/ET should lie between 0.33 and 0.49; these limits fall within the T/ET range of our data (0.41 ± 0.09).

### Comparison of T/ET trends with other products

**Fig. 4:**
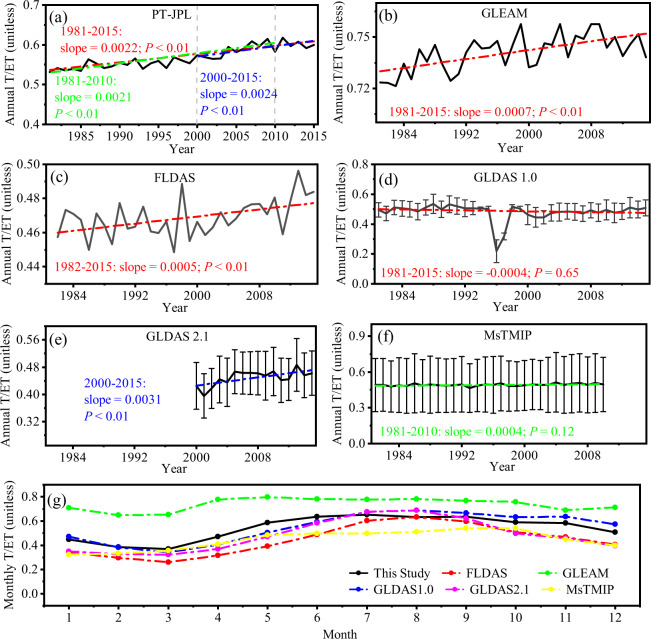
Annual and seasonal T/ET trends acquired from different products. Annual T/ET trends for China according to (**a**) PT-JPL model with optimization parameters, (**b**) GLEAM, (**c**) FLDAS, (**d**) GLDAS 1.0, (**e**) GLDAS 2.1, and (**f**) MsTMIP in mainland China for the period ranging from 1981 to 2015. (**g**) Seasonal variation of T/ET acquired from different products.

We also compared our T/ET dataset with other products, namely GLEAM V3.3^12,104^, FLDAS V1^105^, GLDAS (both V1.0 and V2.1)^106^, and MsTMIP V1^107^ (Supplementary Table S5 shows information on these products). The annual trends of T/ET over China for the period ranging from 1981 to 2015 were analyzed using the Mann-Kendall test and Sen’s Method^108^, as shown in Fig. 4. Out dataset showed a significant increasing trend of annual T/ET over this period at a rate of 0.0022 a^−1^ (*P* < 0.01) for the entire study area (Fig. 4a). Greening can directly explain 57.89% of this T/ET trend^8^. This is consistent with the results at the global scale, i.e., that more than half of the global ET increase since the 1980s can be attributed to greening^19,109^, which is explained by increased transpiration and reduced soil evaporation^9^. Compared with other T/ET products, GLEAM and FLDAS also showed a significant upward trend between the 1980s and 2010s, while their slope values, which were 0.0007 a^−1^ (*P* < 0.01) and 0.0005 a^−1^ (*P* < 0.01), respectively (Fig. 4b and 4c), were lower than those of our simulated results. Meanwhile, the GLDAS 1.0 and MsTMIP products do not reflect the increasing T/ET trend (Fig. 4d, f). The serious discontinuity issues in GLDAS 1.0 forcing data, with large precipitation error in 1996 and temperature errors for the period ranging from 2000 to 2005^110^, may cause the GLDAS 1.0 product failure to reflect the long-term trend of T/ET. Different models of MsTMIP exhibited large differences in the simulated T/ET trends (Supplementary Fig. S6), T/ET acquired from the ISAM model showed a remarkable decreasing trend, with a value of -0.0003 a^−1^, while T/ET from other models all showed remarkable increasing trends, with values between 0.0001 of DLEM and 0.0016 Biome-BGC. After 2000, T/ET from GLDEM 2.1 showed a significant increasing trend, with values of 0.0031 a^−1^ (*P* < 0.01) (Fig. 4e), which was similar to that for our dataset.

We also compared the spatial patterns of the T/ET trend for different products (Supplementary Fig. S7). Our simulated T/ET exhibited finer spatial heterogeneity, compared to the other products. In the vegetation covered area, our simulated T/ET managed to better reflect regional differences; while in the desert area of northwest China, where vegetation cover was scarce, our simulated results correctly showed the relative lower or nearly zero T/ET, while some other products exhibited abnormally high T/ET values.

Figure 4g shows a comparison of the seasonal variation of simulated T/ET with other products. Overall, most of the products exhibited a similar monthly variation, with maximum values being yielded from June to August, and minimum values in March. We further conducted statistical analysis of the correlations between PT-JPL model simulated T/ET and other products at the seasonal scale, the *R*^2^ values exceeded 0.61, and RMSE values were below 38.36% (Supplementary Fig. S8).

### Uncertainties

Model-data fusion is a powerful framework for the generation of improved simulation results via the combination of models with various data streams^21,111^. Observation data, model structure, and model parameters were the three main sources of the uncertainties^111^.

The challenge of acquiring independent observations that represent the scale of measurement is one of the inherent limitations in satellite-based model evaluation^38^. Therefore, we have to acknowledge that the spatial and temporal scale of the observation data used in this study is not the ideal dataset for assessing the performance of PT-JPL model. However, there are only a few alternatives for the calculation and validation of the components of ET (or T/ET). Eddy covariance observations are significantly better for the evaluation of remote-sensing-based evapotranspiration outputs, but they do not provide individual components of evapotranspiration. The sap flow method, which directly reflects the transpiration of single plants, was likely to be smaller in spatial scale than the satellite simulated, however, that method still offers insight into how evapotranspiration should by partitioned. Moreover, the observation data also contained errors stemming from the methodology used, but the field data represented the best available means for the calculation and validation of the remote-sensing-based simulation results^112^.

The PT-JPL model is widely used to simulate evapotranspiration and its individual components based on multiplicative evaporative stress factors^97^, and the model formulation bore similarities with other remote-sensing based evapotranspiration models (e.g., PM-MOD^113^ and GLEAM^12,104^). The simulated evapotranspiration of PT-JPL model was heavily dependent on the accuracy of net radiation^97^, while net radiation had negligible impact on T/ET^8^ because of the linear variation of the evapotranspiration components with net radiation. The simulated evapotranspiration components were generally sensitive to relative humidity and vegetation index^112^. Due to the non-linear relationships between relative humidity and evapotranspiration components (i.e., the model parameter *f*_wet_ was calculated as a function of relative humidity raised to the fourth power), the simulated transpiration components were more sensitive at high relative humidity values than at lower ones. The evapotranspiration partitioning was particularly sensitive to extreme relative humidity^112^. Moreover, bias in PT-JPL due to uncertainties in the vegetation index are consistent with errors found when comparing model estimates to field estimates, meaning that the partitioning of PT-JPL was significantly affected by uncertainty in the vegetation index^112^. Overall, obtaining higher precision forcing data can help improve T/ET simulation results.

Four parameters (*k*_1_, *k*_2_, *β*, and *T*_opt_) were the most sensitive to the model across different biomes^8^. Parameter *β* was more sensitive at low vegetative cover and low precipitation regions, and *T*_opt_ yielded the highest first-order sensitivity indices in the forest and cropland ecosystems. Niu *et al*. (2019) provide further details. In this study, observational data from multiple sites and multiple sources were used to calibrate the parameters of a given ecosystem type, that could better reflect ecological and biophysical properties within an ecosystem. Moreover, compared to the traditional Markov chain Monte Carlo approach, the DEMC algorithm, which was used in this study, is more suitable for drawing inference on high-dimensional models^25,114^.

Overall, despite the uncertainties, the simulated T/ET is consistent with the site observational data. The annual and daily T/ET estimation is close to that of previous studies using a different approach, and the trend of annual T/ET increases are also in line with certain products. Our simulated T/ET showed fine spatial heterogeneity and could accurately reflect the effects of greening on the hydrological cycle.

## Usage Notes

Spatial average T/ET should be calculated as the ratio of regional mean transpiration and regional mean evapotranspiration, instead of directly using the regional or national average values of T/ET. The latter method only reflects the arithmetic mean of T/ET for each pixel, that will strengthen the influence of pixels with lower absolute values of evapotranspiration and transpiration, while weakening the effect of pixels with high transpiration and evapotranspiration.

## Supplementary information

Supplemental information

## Data Availability

The codes for Sobol’ sensitivity analysis, DEMC parameter optimization, PT-JPL model and LAI preparation are available at 10.17605/OSF.IO/MERZN. The codes require MATLAB version 2014a or higher.

## Online-only Table

**Online-only Table 1:** Data organization and descriptions of T/ET, T and ET datasets of China during 1981 to 2015.

Folder	File name	Description	Calibration/Validation
Annual	Annual_T_ET.nc	Name: Ratio of transpiration to evapotranspiration	Type: Validation
		Temporal resolution: Annual	All sites: *R*^2^ = 0.73
		Unit: unitless	RMSE = 0.07 (12.41%)
		Range: 0 – 1	Forest Sites: *R*^2^ = 0.55
		Scale: 1.0	RMSE = 0.08 (13.63%)
		Fill value: -9999	Non-forest sites: *R*^2^ = 0.85
		Missing value: -9999	RMSE = 0.06 (11.41%)
	Annual_T.nc	Name: Transpiration	Type: Calibration
		Temporal resolution: Annual	All sites: *R*^2^ = 0.63
		Unit: mm m^-2^ a^-1^	RMSE = 68.12 (36.80%)
		Range: > 0	Forest Sites: *R*^2^ = 0.65
		Scale: 1.0	RMSE = 75.96 (41.77%)
		Fill value: -9999	Non-forest sites: *R*^2^ = 0.69
		Missing value: -9999	RMSE = 72.46 (37.60%)
	Annual_ET.nc	Name: Evapotranspiration	Type: Calibration
		Temporal resolution: Annual	All sites: *R*^2^ = 0.71
		Unit: mm m^-2^ a^-1^	RMSE = 153.57 (26.21%)
		Valid Range: > 0	Forest Sites: *R*^2^ = 0.68
		Scale Factor: 1.0	RMSE = 147.27 (25.14%)
		No data value: -9999	Non-forest sites: *R*^2^ = 0.64
		Missing value: -9999	RMSE = 116.12 (19.83%)
Daily	Daily_T_ET_YYYY.nc	Name: Ratio of transpiration to evapotranspiration	None
		Temporal resolution: 8-day	
		Unit: unitless	
		Range: 0 – 1	
		Scale: 1.0	
		Fill value: -9999	
		Missing value: -9999	
	Daily_T_YYYY.nc	Name: Transpiration	None
		Temporal resolution: 8-day	
		Unit: mm m^-2^ day^-1^	
		Range: > 0	
		Scale: 1.0	
		Fill value: -9999	
		Missing value: -9999	
	Daily_ET_YYYY.nc	Name: Evapotranspiration	Type: Validation
		Temporal resolution: 8-day	All sites: *R*^2^ = 0.75
		Unit: mm m^-2^ day^-1^	RMSE = 0.62 (35.29%)
		Range: > 0	Forest Sites: *R*^2^ = 0.79
		Scale: 1.0	RMSE = 0.57 (31.07%)
		Fill value: -9999	Non-forest sites: *R*^2^ = 0.71
		Missing value: -9999	RMSE = 0.68 (40.02%)
