# Supplementary material for: A spatial-temporal continuous dataset of the transpiration to evapotranspiration ratio in China from 1981–2015
Source: Sci Data. 2020 Oct 27;7:369. doi: 10.1038/s41597-020-00693-x (PMC7591528; doi:10.1038/s41597-020-00693-x)
Supplement: Supplementary file 1 — Supplemental information [file 41597_2020_693_MOESM1_ESM.docx]

Supplemental information for

**A spatial-temporal continuous dataset of the transpiration to evapotranspiration ratio in China from 1981–2015**

Zhongen Niu^1,2,3^, Honglin He^1,2,4,*^, Gaofeng Zhu^5^, Xiaoli Ren^1,2^, Li Zhang^1,2,4^ Kun Zhang^6^

1. Key Laboratory of Ecosystem Network Observation and Modeling, Institute of Geographic Sciences and Natural Resources Research, Chinese Academy of Sciences, Beijing, 100101, China

2. National Ecosystem Science Data Center, Institute of Geographic Sciences and Natural Resources Research, Chinese Academy of Sciences, Beijing, 100101, China

3. University of Chinese Academy of Sciences, Beijing, 100049, China

4. College of Resources and Environment, University of Chinese Academy of Sciences, Beijing, 100049, China

5. Key Laboratory of Western China’s Environmental Systems (Ministry of Education), College of Earth and Environmental Sciences, Lanzhou University, Lanzhou, 730000, China

6. Institute of Tibetan Plateau Research, Chinese Academy of Sciences, Beijing, 100101, China

corresponding author: Honglin He (hehl@igsnrr.ac.cn)

Table of Contents

[Supplementary Figures 3](#_Toc50126983)

[Fig. S1. Comparison of interpolated relative humidity data with the observed relative humidity data from six ChinaFLUX sites 3](#_Toc50126984)

[Fig. S2. Spatial distribution of annual T/ET, annual transpiration, annual evapotranspiration, and daily evapotranspiration measurement sites 3](#_Toc50126985)

[Fig. S3 Comparison of simulated annual evapotranspiration and transpiration with *in-site* measurements data. 4](#_Toc50126986)

[Fig. S4 Comparison of simulated daily ET with *in-site* measurements data 4](#_Toc50126987)

[Fig. S5. Variation in the 8-day mean value of the simulated ET and the observed ET at the model validation sites 5](#_Toc50126988)

[Fig. S6 Annual T/ET trends of MsTMIP models during 1981–2010 5](#_Toc50126989)

[Fig. S7. The spatial patterns of T/ET trend for different products of China mainland. 6](#_Toc50126990)

[Fig. S8. The relationships between PT-JPL model simulated T/ET and other products at seasonal scale 6](#_Toc50126991)

[Supplementary Tables 7](#_Toc50126992)

[Table S1 Sites Characteristics of 6 ecosystems in ChinaFLUX 7](#_Toc50126993)

[Table S2 Site-scale observational evapotranspiration values extracted from published literature and ChinaFLUX. 7](#_Toc50126994)

[Table S3 Site-scale observational transpiration values extracted from published literature 13](#_Toc50126995)

[Table S4 Site-scale observational T/ET values extracted from published literature. 16](#_Toc50126996)

[Table S5. Information of products which were compared to our simulated T/ET 17](#_Toc50126997)

[References: 18](#_Toc50126998)

# Supplementary Figures





## Fig. S1. Comparison of interpolated relative humidity data with the observed relative humidity data from six ChinaFLUX sites


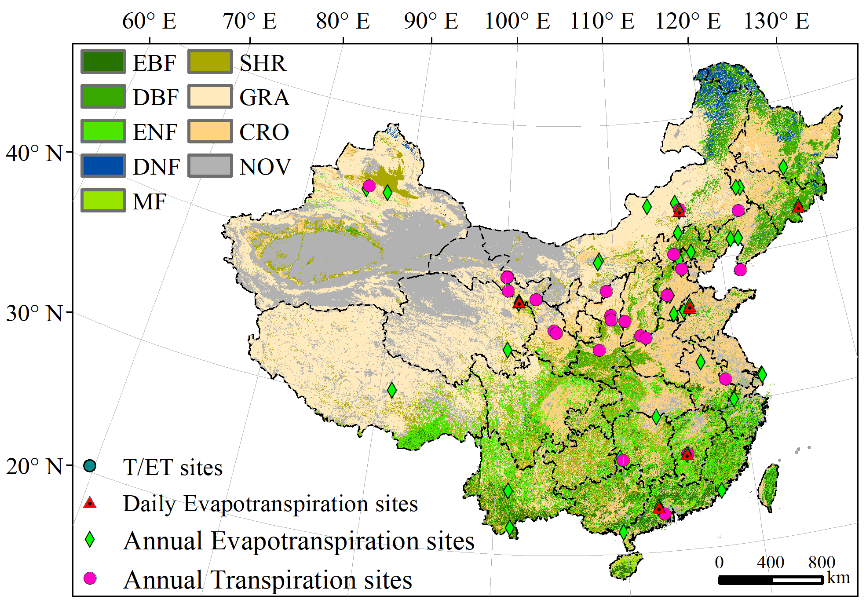


## Fig. S2. Spatial distribution of annual T/ET, annual transpiration, annual evapotranspiration, and daily evapotranspiration measurement sites. The base map reflects vegetation cover in mainland China. EBF = evergreen broadleaf forest; DBF = deciduous broadleaf forest; ENF = evergreen needleleaf forest; DNF = deciduous needleleaf forest; MF = mixed forest; SHR = shrubland; GRA = grassland; and CRO = cropland. The picture was reprint from Niu et al., (2019).





## Fig. S3 Comparison of simulated annual evapotranspiration and transpiration with *in-site* measurements data. The picture was reprinted from Niu et al., (2019).





## Fig. S4 Comparison of simulated daily ET with *in-site* measurements data

~~

~~

## Fig. S5. Variation in the 8-day mean value of the simulated ET and the observed ET at the model validation sites. EBF = evergreen broadleaf forest; ENF = evergreen needleleaf forest; MF = mixed forest; SHR = shrubland; GRA = grassland; and CRO = cropland





## Fig. S6 Annual T/ET trends of MsTMIP models during 1981–2010


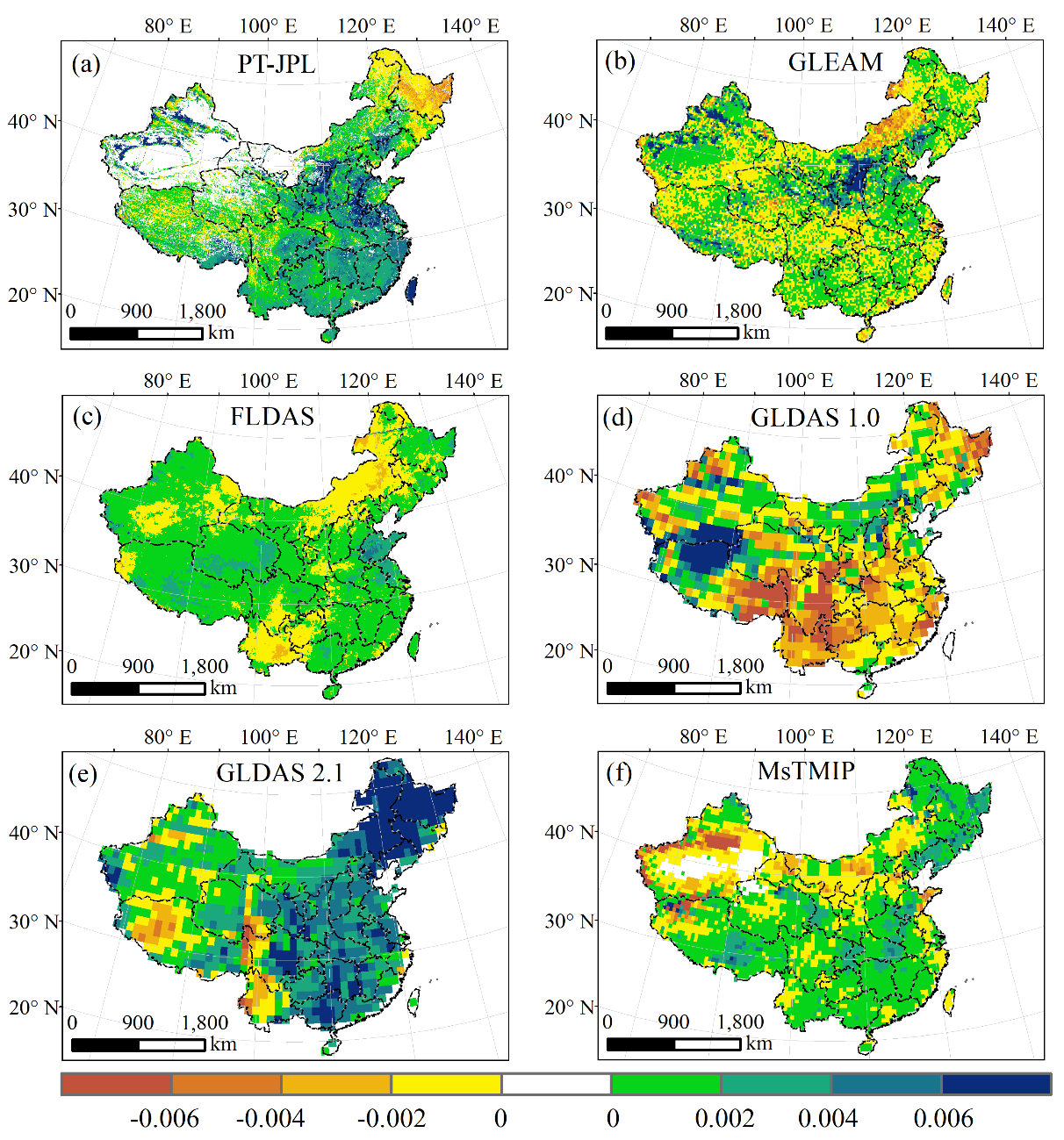


## Fig. S7. The spatial patterns of T/ET trend for different products of China mainland.





## Fig. S8. The relationships between PT-JPL model simulated T/ET and other products at seasonal scale

# Supplementary Tables

## Table S1 Sites Characteristics of 6 ecosystems in ChinaFLUX

| No1. | Site name | Vegetation type | Lat (°E) | Lon (°N) | duration |
| --- | --- | --- | --- | --- | --- |
| 1 | DHS | EBF (Forest) | 23.17 | 112.53 | 2003–2008 |
| 2 | QYZ | ENF (Forest) | 26.73 | 115.05 | 2003–2008 |
| 3 | MF | MF (Forest) | 42.40 | 128.10 | 2003–2008 |
| 4 | HBGC | SHR (Shrub) | 37.67 | 101.33 | 2003–2008 |
| 5 | NM | GRA (Grassland) | 43.33 | 116.40 | 2004–2008 |
| 6 | YC | CRO (Cropland) | 36.83 | 116.57 | 2003–2008 |

EBF = Evergreen broadleaf forest, ENF = Evergreen needleleaf forest, MF = Mixed forest; SHR = shrub; GRA = grassland; CRO = cropland

## Table S2 Site-scale observational evapotranspiration values extracted from published literature and ChinaFLUX.

| No. | Site Name | Lat (°E) | Lon (°N) | Altitude(m) | Vegetation Type | MAT(°C) | MAP(mm) | duration | Observed ET | Simulated ET | Reference |
| --- | --- | --- | --- | --- | --- | --- | --- | --- | --- | --- | --- |
| 1 | DXF | 39.53 | 116.25 | 30 | Forest | 12.53 | 482.00 | 2006 | 599.00 | 407.96 | Zhang, 2010 |
|  | DXF | 39.53 | 116.25 | 30 | Forest | 13.21 | 661.00 | 2007 | 561.60 | 416.61 |  |
|  | DXF | 39.53 | 116.25 | 30 | Forest | 13.62 | 662.00 | 2008 | 672.47 | 465.35 |  |
|  | DXF | 39.53 | 116.25 | 30 | Forest | 12.65 | 414.00 | 2009 | 517.00 | 396.23 |  |
| 2 | LS | 45.33 | 127.57 | 340 | Forest | 2.80 | 700.00 | 2004 | 328.53 | 458.68 | Cui, 2007 |
|  | LS | 45.33 | 127.57 | 340 | Forest | 2.80 | 700.00 | 2005 | 363.17 | 421.85 |  |
|  | LS | 45.33 | 127.57 | 340 | Forest | 2.80 | 700.00 | 2006 | 284.48 | 439.74 |  |
| 3 | GT | 38.53 | 100.25 | 2835 | Forest | 0.94 | 428.00 | 2011 | 417.00 | 268.15 | Zhu et al., 2014 |
| 4 | QYZ | 26.73 | 115.05 | 110.8 | Forest | 18.74 | 949.00 | 2003 | 787.22 | 864.42 | ChinaFLUX |
|  | QYZ | 26.73 | 115.05 | 110.8 | Forest | 18.26 | 1375.50 | 2004 | 867.22 | 828.97 |  |
|  | QYZ | 26.73 | 115.05 | 110.8 | Forest | 17.63 | 1455.40 | 2005 | 594.54 | 821.73 |  |
|  | QYZ | 26.73 | 115.05 | 110.8 | Forest | 18.03 | 1485.30 | 2006 | 722.37 | 808.97 |  |
|  | QYZ | 26.73 | 115.05 | 110.8 | Forest | 18.50 | 1318.70 | 2007 | 895.22 | 802.46 |  |
|  | QYZ | 26.73 | 115.05 | 110.8 | Forest | 17.95 | 1332.90 | 2008 | 862.97 | 783.96 |  |
|  | QYZ | 26.73 | 115.05 | 110.8 | Forest | 18.33 | 1265.30 | 2009 | 900.33 | 830.14 |  |
|  | QYZ | 26.73 | 115.05 | 110.8 | Forest | 17.80 | 1854.30 | 2010 | 839.81 | 768.74 |  |
|  | QYZ | 26.73 | 115.05 | 110.8 | Forest | 17.32 | 1237.30 | 2011 | 816.87 | 711.17 |  |
| 5 | CBS | 42.40 | 128.10 | 738 | Forest | 4.66 | 538.40 | 2003 | 454.05 | 404.39 | ChinaFLUX |
|  | CBS | 42.40 | 128.10 | 738 | Forest | 4.88 | 713.10 | 2004 | 539.81 | 408.83 |  |
|  | CBS | 42.40 | 128.10 | 738 | Forest | 3.35 | 924.20 | 2005 | 513.79 | 372.82 |  |
|  | CBS | 42.40 | 128.10 | 738 | Forest | 4.28 | 656.70 | 2006 | 573.68 | 397.86 |  |
|  | CBS | 42.40 | 128.10 | 738 | Forest | 4.95 | 822.40 | 2007 | 534.60 | 389.86 |  |
|  | CBS | 42.40 | 128.10 | 738 | Forest | 4.73 | 558.90 | 2008 | 496.63 | 404.78 |  |
|  | CBS | 42.40 | 128.10 | 738 | Forest | 4.15 | 792.00 | 2009 | 502.27 | 382.37 |  |
|  | CBS | 42.40 | 128.10 | 738 | Forest | 3.60 | 886.40 | 2010 | 513.01 | 403.04 |  |
| 675 | DHS | 23.17 | 112.53 | 300 | Forest | 20.67 | 1289.40 | 2003 | 758.51 | 960.57 | ChinaFLUX |
|  | DHS | 23.17 | 112.53 | 300 | Forest | 20.49 | 1297.50 | 2004 | 781.19 | 914.23 |  |
|  | DHS | 23.17 | 112.53 | 300 | Forest | 20.05 | 1615.00 | 2005 | 721.71 | 853.53 |  |
|  | DHS | 23.17 | 112.53 | 300 | Forest | 20.53 | 2227.60 | 2006 | 674.16 | 860.31 |  |
|  | DHS | 23.17 | 112.53 | 300 | Forest | 20.62 | 1423.10 | 2007 | 723.36 | 927.60 |  |
|  | DHS | 23.17 | 112.53 | 300 | Forest | 19.89 | 2267.30 | 2008 | 697.29 | 854.21 |  |
|  | DHS | 23.17 | 112.53 | 300 | Forest | 20.48 | 1760.40 | 2009 | 799.24 | 867.05 |  |
|  | DHS | 23.17 | 112.53 | 300 | Forest | 19.81 | 1735.90 | 2010 | 709.97 | 856.46 |  |
| 7 | THY | 30.18 | 119.34 | 185 | Forest | 16.00 | 1201.72 | 2011 | 669.84 | 597.02 | Lin et al., 2013 |
| 8 | YY | 29.53 | 112.86 | 31 | Forest | 16.75 | 1500.63 | 2006 | 727.06 | 891.71 | Wang, 2008 |
| 9 | HT | 26.83 | 109.75 | 330 | Forest | 16.80 | 1107.00 | 2009 | 709.40 | 667.47 | Wang et al., 2011 |
| 10 | XLD | 35.02 | 112.47 | 410 | Forest | 15.40 | 505.00 | 2007 | 555.01 | 435.64 | Guo, 2010 |
|  | XLD | 35.02 | 112.47 | 410 | Forest | 14.80 | 333.00 | 2008 | 523.43 | 444.75 |  |
|  | XLD | 35.02 | 112.47 | 410 | Forest | 14.70 | 325.00 | 2009 | 591.53 | 435.75 |  |
| 11 | XSBN | 21.93 | 101.27 | 750 | Forest | 19.42 | 1247.00 | 2003 | 1052.00 | 1108.03 | Li et al., 2010 |
|  | XSBN | 21.93 | 101.27 | 750 | Forest | 19.24 | 1428.00 | 2004 | 1027.00 | 924.39 |  |
|  | XSBN | 21.93 | 101.27 | 750 | Forest | 19.41 | 1284.00 | 2005 | 1048.00 | 976.96 |  |
|  | XSBN | 21.93 | 101.27 | 750 | Forest | 19.48 | 1328.00 | 2006 | 988.00 | 1026.86 |  |
| 12 | ALS | 24.53 | 101.02 | 2476 | Forest | 11.96 | 1364.10 | 2010 | 803.03 | 651.45 | Tan et al., 2011a |
| 13 | KBQF | 40.54 | 108.69 | 1033 | Forest | 6.30 | 318.00 | 2006 | 351.63 | 242.97 | Wilske et al., 2009 |
| 14 | HN | 33.00 | 117.00 | 15 | Forest | 17.67 | 1500.00 | 2005 | 918.77 | 591.40 | Han et al., 2009 |
|  | HN | 33.00 | 117.00 | 15 | Forest | 18.06 | 1500.00 | 2006 | 1012.91 | 626.75 |  |
| 15 | MY | 40.63 | 117.32 | 350 | Forest | 9.73 | 562.60 | 2008 | 582.07 | 420.38 | Liu et al., 2013 |
|  | MY | 40.63 | 117.32 | 350 | Forest | 9.60 | 564.50 | 2009 | 602.07 | 392.63 |  |
|  | MY | 40.63 | 117.32 | 350 | Forest | 8.70 | 640.50 | 2010 | 626.72 | 423.15 |  |
| 16 | CP | 40.17 | 116.13 | 61.7 | Forest | 11.90 | 473.50 | 2011 | 790.60 | 590.63 | Ouyang et al., 2013 |
| 17 | ZC | 37.80 | 114.93 | 40 | Forest | 12.90 | 507.00 | 2012 | 759.00 | 444.78 | Zhang et al., 2013 |
| 18 | ML | 21.93 | 101.27 | 750 | Forest | 21.70 | 1504.00 | 2008 | 1125.00 | 905.65 | Tan et al., 2011b |
| 19 | FK | 44.28 | 87.93 | 475 | Shrub | 6.50 | 173.20 | 2004 | 205.00 | 201.41 | Liu et al., 2012 |
| 20 | KBQ | 40.38 | 108.55 | 1169.2 | Shrub | 6.30 | 318.00 | 2006 | 329.77 | 229.03 | Wilske et al., 2009 |
| 21 | HBGC | 37.67 | 101.33 | 3293 | Shrub | -1.40 | 546.10 | 2003 | 503.20 | 311.88 | ChinaFLUX |
|  | HBGC | 37.67 | 101.33 | 3293 | Shrub | -1.94 | 493.50 | 2004 | 499.79 | 289.75 |  |
|  | HBGC | 37.67 | 101.33 | 3293 | Shrub | -1.33 | 448.40 | 2005 | 499.72 | 304.70 |  |
|  | HBGC | 37.67 | 101.33 | 3293 | Shrub | -1.14 | 565.20 | 2006 | 573.65 | 298.74 |  |
|  | HBGC | 37.67 | 101.33 | 3293 | Shrub | -1.32 | 510.00 | 2007 | 681.28 | 497.76 |  |
|  | HBGC | 37.67 | 101.33 | 3293 | Shrub | -1.85 | 429.90 | 2008 | 572.23 | 280.01 |  |
|  | HBGC | 37.67 | 101.33 | 3293 | Shrub | -0.62 | 494.60 | 2009 | 499.01 | 291.10 |  |
|  | HBGC | 37.67 | 101.33 | 3293 | Shrub | -0.83 | 493.30 | 2010 | 451.59 | 321.22 |  |
|  | HBGC | 37.67 | 101.33 | 3293 | Shrub | -1.53 | 524.40 | 2011 | 469.81 | 290.49 |  |
| 22 | DLC | 42.05 | 116.28 | 1350 | Cropland | 2.73 | 424.00 | 2006 | 394.80 | 321.03 | Chen et al., 2009 |
| 23 | TYC | 44.57 | 122.92 | 184 | Cropland | 6.70 | 333.00 | 2003 | 338.30 | 345.55 | Liu and Feng, 2012 |
|  | TYC | 44.57 | 122.92 | 184 | Cropland | 7.00 | 209.00 | 2004 | 222.20 | 310.65 |  |
|  | TYC | 44.57 | 122.92 | 184 | Cropland | 5.70 | 334.00 | 2005 | 340.30 | 380.81 |  |
|  | TYC | 44.57 | 122.92 | 184 | Cropland | 6.30 | 285.00 | 2006 | 295.30 | 328.58 |  |
|  | TYC | 44.57 | 122.92 | 184 | Cropland | 7.60 | 194.00 | 2007 | 213.10 | 302.14 |  |
|  | TYC | 44.57 | 122.92 | 184 | Cropland | 7.10 | 421.00 | 2008 | 428.80 | 349.81 |  |
| 24 | WS | 36.65 | 116.05 | 30 | Cropland | 13.60 | 360.00 | 2006 | 619.50 | 576.61 | Shen et al., 2013 |
|  | WS | 36.65 | 116.05 | 30 | Cropland | 13.90 | 480.00 | 2007 | 595.00 | 610.25 |  |
|  | WS | 36.65 | 116.05 | 30 | Cropland | 13.70 | 566.00 | 2009 | 666.00 | 613.12 |  |
| 25 | YC | 36.83 | 116.57 | 28 | Cropland | 12.94 | 696.30 | 2003 | 559.74 | 522.27 | ChinaFLUX |
|  | YC | 36.83 | 116.57 | 28 | Cropland | 13.59 | 836.50 | 2004 | 630.54 | 513.84 |  |
|  | YC | 36.83 | 116.57 | 28 | Cropland | 13.05 | 678.40 | 2005 | 608.66 | 496.08 |  |
|  | YC | 36.83 | 116.57 | 28 | Cropland | 13.67 | 381.80 | 2006 | 620.46 | 493.84 |  |
|  | YC | 36.83 | 116.57 | 28 | Cropland | 13.74 | 571.70 | 2007 | 648.90 | 523.09 |  |
|  | YC | 36.83 | 116.57 | 28 | Cropland | 13.40 | 527.10 | 2008 | 653.61 | 537.76 |  |
|  | YC | 36.83 | 116.57 | 28 | Cropland | 13.25 | 759.10 | 2009 | 671.13 | 503.54 |  |
|  | YC | 36.83 | 116.57 | 28 | Cropland | 13.11 | 739.90 | 2010 | 658.43 | 523.45 |  |
|  | YC | 36.83 | 116.57 | 28 | Cropland | 12.69 | 565.90 | 2011 | 652.78 | 412.82 |  |
| 26 | PJC | 41.15 | 121.92 | 3.8 | Cropland | 8.60 | 631.00 | 2006 | 725.64 | 483.52 | Zhou and Wang, 2010 |
| 27 | LC | 37.83 | 114.67 | 50 | Cropland | 12.80 | 529.00 | 2009 | 675.00 | 655.39 | Shen et al., 2013 |
|  | LC | 37.83 | 114.67 | 50 | Cropland | 12.80 | 366.00 | 2010 | 687.00 | 633.27 |  |
|  | LC | 37.83 | 114.67 | 50 | Cropland | 12.80 | 370.00 | 2011 | 717.00 | 552.21 |  |
| 28 | JZ | 41.15 | 121.20 | 17 | Cropland | 8.50 | 568.80 | 2006 | 451.97 | 498.56 | Zhou and Wang, 2010 |
| 29 | DXC | 39.62 | 116.43 | 20 | Cropland | 12.89 | 561.93 | 2008 | 650.30 | 467.73 | Liu et al., 2013 |
|  | DXC | 39.62 | 116.43 | 20 | Cropland | 12.64 | 404.30 | 2009 | 729.29 | 404.23 |  |
|  | DXC | 39.62 | 116.43 | 20 | Cropland | 12.39 | 372.90 | 2010 | 614.55 | 421.74 |  |
| 30 | GTC | 36.52 | 115.13 | 30 | Cropland | 14.12 | 457.70 | 2008 | 515.95 | 551.76 | Gao et al., 2013 |
|  | GTC | 36.52 | 115.13 | 30 | Cropland | 13.80 | 435.86 | 2009 | 645.74 | 506.85 |  |
|  | GTC | 36.52 | 115.13 | 30 | Cropland | 13.54 | 577.60 | 2010 | 664.27 | 519.47 |  |
| 31 | WLWS | 44.28 | 85.82 | 469 | Cropland | 8.40 | 216.00 | 2010 | 560.00 | 323.09 | Zhou et al., 2012 |
| 32 | DLG | 42.05 | 116.28 | 1350 | Grassland | 2.73 | 424.00 | 2006 | 433.53 | 302.67 | Chen et al., 2009 |
| 33 | XLHG | 43.55 | 116.67 | 1250 | Grassland | 1.51 | 202.00 | 2006 | 281.57 | 244.50 | Chen et al., 2009 |
| 34 | XFS | 44.13 | 116.33 | 1030 | Grassland | 2.61 | 243.87 | 2004 | 323.14 | 231.18 | Zheng et al., 2016 |
|  | XFS | 44.13 | 116.33 | 1030 | Grassland | 1.48 | 159.37 | 2005 | 219.98 | 226.55 |  |
|  | XFS | 44.13 | 116.33 | 1030 | Grassland | 1.86 | 224.92 | 2006 | 318.04 | 211.52 |  |
| 35 | HB | 37.60 | 101.30 | 3250 | Grassland | -1.20 | 554.00 | 2002 | 341.00 | 329.62 | Gu et al., 2008 |
|  | HB | 37.60 | 101.30 | 3250 | Grassland | -0.70 | 705.60 | 2003 | 407.00 | 325.30 |  |
|  | HB | 37.60 | 101.30 | 3250 | Grassland | -1.60 | 666.20 | 2004 | 426.00 | 302.92 |  |
| 36 | TYG | 44.59 | 122.52 | 184 | Grassland | 6.70 | 333.00 | 2003 | 323.60 | 283.93 | Liu and Feng, 2012 |
|  | TYG | 44.59 | 122.52 | 184 | Grassland | 7.00 | 209.00 | 2004 | 201.50 | 248.89 |  |
|  | TYG | 44.59 | 122.52 | 184 | Grassland | 5.70 | 334.00 | 2005 | 330.00 | 323.44 |  |
|  | TYG | 44.59 | 122.52 | 184 | Grassland | 6.30 | 285.00 | 2006 | 287.00 | 262.60 |  |
|  | TYG | 44.59 | 122.52 | 184 | Grassland | 7.60 | 194.00 | 2007 | 193.70 | 232.41 |  |
|  | TYG | 44.59 | 122.52 | 184 | Grassland | 7.10 | 421.00 | 2008 | 412.90 | 296.56 |  |
| 37 | DX | 30.85 | 91.08 | 4333 | Grassland | 1.80 | 550.40 | 2004 | 606.65 | 373.06 | ChinaFLUX |
|  | DX | 30.85 | 91.08 | 4333 | Grassland | 2.53 | 487.00 | 2005 | 486.58 | 380.89 |  |
|  | DX | 30.85 | 91.08 | 4333 | Grassland | 3.03 | 394.50 | 2006 | 481.86 | 331.58 |  |
|  | DX | 30.85 | 91.08 | 4333 | Grassland | 2.98 | 487.00 | 2007 | 487.58 | 291.59 |  |
|  | DX | 30.85 | 91.08 | 4333 | Grassland | 2.35 | 593.70 | 2008 | 617.74 | 332.39 |  |
|  | DX | 30.85 | 91.08 | 4333 | Grassland | 3.45 | 266.00 | 2009 | 457.68 | 301.03 |  |
|  | DX | 30.85 | 91.08 | 4333 | Grassland | 3.24 | 379.60 | 2010 | 566.34 | 304.30 |  |
| 38 | XLG | 43.53 | 116.67 | 1200 | Grassland | 1.96 | 364.40 | 2004 | 365.09 | 257.38 | ChinaFLUX |
|  | XLG | 43.53 | 116.67 | 1200 | Grassland | 0.83 | 154.60 | 2005 | 291.31 | 254.69 |  |
|  | XLG | 43.53 | 116.67 | 1200 | Grassland | 1.54 | 304.10 | 2006 | 307.70 | 248.08 |  |
|  | XLG | 43.53 | 116.67 | 1200 | Grassland | 2.25 | 240.00 | 2007 | 346.95 | 207.10 |  |
|  | XLG | 43.53 | 116.67 | 1200 | Grassland | 1.61 | 362.20 | 2008 | 354.65 | 257.94 |  |
|  | XLG | 43.53 | 116.67 | 1200 | Grassland | 1.13 | 281.40 | 2009 | 295.27 | 217.89 |  |
|  | XLG | 43.53 | 116.67 | 1200 | Grassland | 0.82 | 318.60 | 2010 | 391.03 | 256.97 |  |
|  | XLG | 43.53 | 116.67 | 1200 | Grassland | 0.69 | 286.70 | 2011 | 415.12 | 279.58 |  |
| 39 | SJY | 34.35 | 100.50 | 3963 | Grassland | 0.18 | 460.70 | 2006 | 452.24 | 327.84 | Li et al., 2012 |
|  | SJY | 34.35 | 100.50 | 3963 | Grassland | 0.64 | 496.10 | 2007 | 474.24 | 309.46 |  |
|  | SJY | 34.35 | 100.50 | 3963 | Grassland | -0.15 | 480.10 | 2008 | 459.57 | 313.08 |  |

## Table S3 Site-scale observational transpiration values extracted from published literature. The table was first published in Niu et al. (2019)

| No. | Site name | Lat  (°N) | Lon  (°E) | Vegetation types | MAT (°C) | MAP (mm) | years | Duration/  Month | Observation Transpiration /mm | Simualted Transpiration /mm | Reference |
| --- | --- | --- | --- | --- | --- | --- | --- | --- | --- | --- | --- |
| 1 | Heshan | 22.68 | 112.90 | EBF | - | 1122.4 | 2004 | 1-12 | 244.50 | 128.38 | Ma et al., 2008 |
|  |  |  |  |  |  |  | 2005 | 1-12 | 185.80 | 115.54 |  |
| 2 | Huitong | 26.83 | 109.75 | EBF | 16.8 | 1268 | 2015 | 1-12 | 522.1 | 531.24 | Ouyang et al., 2018 |
| 3 | Daqinggou | 42.97 | 122.35 | ENF | 6.4 | 316.7 | 2011 | 5-9 | 20.90 | 28.58 | Song et al., 2018 |
|  |  |  |  |  |  |  | 2012 | 5-9 | 22.90 | 28.95 |  |
| 4 | Longtan | 35.74 | 104.48 | ENF | 6.8 | 386 | 2015 | 5-9 | 97.95 | 28.43 | Zhang et al., 2017 |
|  |  |  |  |  |  |  | 2016 | 5-9 | 122.85 | 35.83 |  |
| 5 | Anjiapo | 35.57 | 104.66 | ENF | - | 327.6 | 2014 | 4-9 | 138.6 | 22.32 | Zhang et al., 2017 |
|  |  |  |  |  |  |  | 2015 | 4-9 | 107.60 | 31.73 |  |
| 6 | Pailougou | 38.40 | 100.28 | ENF | 0.5 | 378.6 | 2011 | 6-9 | 195.20 | 71.11 | Chang et al., 2014 |
|  |  |  |  |  |  |  | 2012 | 6-9 | 219.60 | 61.37 |  |
| 7 | Anjiagou | 35.58 | 104.65 | ENF | 8.5 | 421 | 2014 | 5-9 | 277.68 | 115.00 | Fang et al., 2016 |
| 8 | Qianyanzhou | 26.75 | 115.07 | ENF | 17.9 | 1324 | 2004 | 1-12 | 477.00 | 457.39 | Liu et al., 2008 |
| 9 | Manasi | 44.50 | 86.00 | DBF | 6.9 | 190.3 | 2014 | 1-12 | 55.67 | 71.75 | Fu et al., 2017 |
| 10 | Yangjuangou | 36.70 | 109.52 | DBF | 9.8 | 531 | 2013 | 5-9 | 21.00 | 49.36 | Jiao et al., 2016 |
|  |  |  |  |  |  |  | 2014 | 5-9 | 36.00 | 98.79 |  |
| 11 | Gonglushan | 36.42 | 109.53 | DBF | 10.6 | 498 | 2008 | 4-10 | 92.30 | 213.50 | Zhang et al., 2015 |
|  |  |  |  |  |  |  | 2009 | 4-10 | 61.70 | 184.84 |  |
| 12 | Gonglushan | 36.42 | 109.53 | DBF | 10.1 | 526 | 2008 | 4-10 | 106.47 | 213.50 | Yan et al., 2016 |
|  |  |  |  |  |  |  | 2009 | 4-10 | 83.30 | 184.84 |  |
|  |  |  |  |  |  |  | 2010 | 4-10 | 75.27 | 176.60 |  |
| 13 | Daxing | 39.53 | 116.25 | DBF | 11.5 | 404.8 | 2010 | 5-10 | 113.70 | 67.41 | Mo et al., 2014 |
|  |  |  |  |  |  |  | 2011 | 5-10 | 174.80 | 71.27 |  |
| 14 | Linze | 39.32 | 100.10 | DBF | 7.6 | 116.8 | 2006 | 5-10 | 155.52 | 151.32 | Zhao et al., 2009 |
| 15 | Taihangshan | 35.18 | 112.05 | DBF | - | 641.7 | 2005 | 1-12 | 426.20 | 363.12 | Gao et al., 2009 |
|  |  |  |  |  |  |  | 2006 | 1-12 | 362.80 | 353.10 |  |
| 16 | Guanting | 40.58 | 115.67 | DBF | 12.2 | 549.8 | 2007 | 4-9 | 488.02 | 460.78 | Ren et al., 2008 |
| 17 | Xiaolangdi | 35.02 | 112.47 | DBF | - | 641.7 | 2005 | 4-9 | 265.30 | 298.19 | Wang et al., 2007 |
|  |  |  |  |  |  |  | 2006 | 4-9 | 194.90 | 312.23 |  |
| 18 | Dongshanqiao | 31.62 | 118.85 | DBF | 18.06 | 1100 | 2013 | 5-10 | 237.52 | 274.80 | Liu et al., 2015 |
| 19 | Caijiachuan | 36.28 | 110.72 | MF | 10 | 579 | 2008 | 7-10 | 51.39 | 133.83 | Chen et al., 2014 |
|  |  |  |  |  |  |  | 2009 | 7-10 | 47.31 | 143.08 |  |
|  |  |  |  |  |  |  | 2010 | 7-10 | 51.07 | 138.89 |  |
| 20 | Laodong | 38.90 | 121.62 | MF | 10.2 | 234.7 | 2008 | 7-10 | 154.01 | 208.49 | Chen et al., 2011 |
|  |  |  |  |  |  |  | 2009 | 7-10 | 174.01 | 286.12 |  |
| 21 | LIRBRS | 39.37 | 100.15 | SHR | - | 123.5 | 2014 | 5-10 | 140.78 | 156.97 | Ji et al., 2016 |
|  |  |  |  |  |  |  | 2008-2010 | 5-10 | 166.00 | 110.94 |  |
| 22 | - | 37.87 | 102.83 | SHR | - | 164.4 | 2013 | 5-10 | 173.00 | 166.91 | Zhao et al., 2018 |
|  |  |  |  |  |  |  | 2014 | 5-10 | 167.00 | 167.63 |  |
|  |  |  |  |  |  |  | 2015 | 5-10 | 158.00 | 204.58 |  |
| 23 | IMGERS | 43.63 | 116.71 | GRA | 0.7 | 343 | 2004 | 1-12 | 155.25 | 73.756 | Chen et al., 2007 |
|  |  |  |  |  |  |  | 2005 | 1-12 | 60.43 | 52.47 |  |
|  |  |  |  |  |  |  | 2006 | 1-12 | 95.70 | 70.17 |  |
| 24 | Shiyanghe | 37.87 | 102.83 | CRO | - | 68.4 | 2013 | 4-9 | 315.54 | 362.75 | Jiang et al., 2016 |
|  |  |  |  |  |  | 206.2 | 2014 | 4-9 | 321.95 | 361.91 |  |
| 25 | Zhangye | 39.35 | 100.12 | CRO | - | 386 | 2015 | 5-10 | 200.00 | 58.71 | Gao et al., 2018 |
|  |  |  |  |  |  | 461 | 2016 | 5-10 | 197.00 | 52.62 |  |
| 26 | Bulang | 38.39 | 109.20 | CRO | - | 340 | 2011 | 5-9 | 245.00 | 85.14 | Hou et al., 2014 |

* All the transpiration data were determined by the sap flow method except for the grassland site, which was inversed by Hydrus-1D model

## Table S4 Site-scale observational T/ET values extracted from published literature. Part of the table was first published in Niu et al. (2019)

| ID | Ecoregion | Lat (°E) | Lon (°N) | Period | Method | Observation T/ET | Estimated T/ET | References |
| --- | --- | --- | --- | --- | --- | --- | --- | --- |
| 1 | Forest | 21.00 | 109.90 | Sep 1999 – Sep 2000 | sap flow, eddy covariance | 0.57 | 0.48 | Zhou et al., 2004 |
| 2 | Forest | 23.20 | 112.50 | 2003–2011 for ET, July 2010–June 2011 for T | eddy covariance, sap flow probes | 0.60 | 0.61 | Liu et al., 2015 |
| 3 | Forest | 42.40 | 128.09 | 2003–2009 | eddy covariance | 0.56 | 0.70 | Zhu et al., 2015 |
| 4 | Forest | 26.73 | 115.05 | 2004–2006 | eddy covariance | 0.77 | 0.77 | Zhu et al., 2015 |
| 5 | Forest | 23.17 | 112.53 | 2003–2006 | eddy covariance | 0.72 | 0.73 | Zhu et al., 2015 |
| 6 | Forest | 23.17 | 112.52 | 2010–2011 | sap flow and eddy convergence | 0.60 | 0.73 | Liu et al., 2015 |
| 7 | Forest | 38.53 | 100.25 | 2008 | modified Penman–Monteith equation | 0.51 | 0.44 | Tian et al., 2011 |
| 8 | Shrubland | 39.40 | 100.10 | 2008–2010 | Sap flow | 0.76 | 0.68 | Zhao et al., 2016 |
| 9 | Shrubland | 38.60 | 103.50 | May 2010–June 2012 | Bowen ratio energy balance and water balance, thermal infrared remote sensing | 0.69 | 0.64 | Qiu et al., 2015 |
| 10 | Cropland | 37.87 | 102.85 | 2009 | sap flow and micro-lysimetry techniques | 0.52 | 0.50 | Zhang et al., 2011 |
| 11 | Cropland | 35.28 | 107.88 | July–September 2012 | sap flow | 0.63 | 0.59 | Zhou et al., 2017 |
| 12 | Cropland | 37.75 | 113.20 | 2015 | water vapor and energy flux observations | 0.59 | 0.64 | Gao et al., 2018 |
| 13 | Grassland | 37.60 | 101.70 | 2003–2005 | model (with meteorological data) | 0.39 | 0.38 | Hu et al., 2009 |
| 14 | Grassland | 37.70 | 101.70 | 2003–2005 | model (with meteorological data) | 0.37 | 0.37 | Hu et al., 2009 |
| 15 | Grassland | 30.90 | 91.10 | 2003–2005 | model (with meteorological data) | 0.56 | 0.43 | Hu et al., 2009 |
| 16 | Grassland | 43.60 | 116.70 | 2003–2005 | model (with meteorological data) | 0.44 | 0.42 | Hu et al., 2009 |
| 17 | Grassland | 43.50 | 116.70 | May–September 2003 | model (with meteorological data) | 0.62 | 0.51 | Huang et al., 2010 |
| 18 | Grassland | 44.30 | 87.90 | May–October | energy balance model | 0.38 | 0.42 | Liu et al., 2012 |

## Table S5. Information of products which were compared to our simulated T/ET

| ID | Product Name | Model | Temporal resolution | Spatial resolution | Period | Reference URL |
| --- | --- | --- | --- | --- | --- | --- |
| 1 | GLEAM V3.3 | GLEAM | 1 month | 0.25°×0.25° | 1981–2015 | <https://www.gleam.eu/> |
| 2 | FLDAS | NOAH | 1 month | 0.01°×0.01° | 1982–2015 | <https://ldas.gsfc.nasa.gov/index.php/fldas> |
| 3 | GLDAS V1.0 | CLM, MOS, NOAH | 1 month | 1°×1° | 1981–2015 | <https://ldas.gsfc.nasa.gov/index.php/gldas> |
| 4 | GLDAS V2.1 | CLSM, NOAH, VIC | 1 month | 1°×1° | 2000–2015 | <https://ldas.gsfc.nasa.gov/index.php/gldas> |
| 5 | MsTMIP | CLM4, CLM4VIC, DLEM, BIOME-BGC, CLASS-CTEM-N, and ISAM | 1 month | 0.5°×0.5° | 1981–2010 | <https://daac.ornl.gov/NACP/guides/NACP_MsTMIP_TBMO.html> |

# References:

Chang, X., Zhao, W., Liu, H., Wei, X., Liu, B. & He, Z. Qinghai spruce (Picea crassifolia) forest transpiration and canopy conductance in the upper Heihe River Basin of arid northwestern China. *Agr. Forest Meteorol.* **198–199**, 209–220 (2014).

Chen, L. *et al*. Response of transpiration to rain pulses for two tree species in a semiarid plantation. *Int. J. Biometeorol.* **58**, 1569–1581 (2014).

Chen, L., Zhang, Z., Li, Z., Tang, J., Caldwell, P. & Zhang, W. Biophysical control of whole tree transpiration under an urban environment in Northern China. *J. Hydrol.* **402**, 388–400 (2011).

Chen, S. *et al*. Energy balance and partition in Inner Mongolia steppe ecosystems with different land use types. *Agr. Forest Meteorol.* **149**, 1800–1809 (2009).

Chen, Y., Lee, G., Lee, P. & Oikawa, T. Model analysis of grazing effect on above-ground biomass and above-ground net primary production of a Mongolian grassland ecosystem. *J. Hydrol.* **333**, 155–164 (2007).

Coenders-Gerrits, A. M. *et al*. Uncertainties in transpiration estimates. *Nature* **506**, E1–2 (2014)

Cui, S. *Study on the CO2 flux of a larch plantation in NE China by the micrometeorological method* (Northeast Forestry University, 2007). (in Chinese)

Fang, S., Zhao, C. & Jian, S. Canopy transpiration of Pinus tabulaeformis plantation forest in the Loess Plateau region of China. *Environ. Earth Sci.* **75**, 9 (2016).

Fu, S., Sun, L. & Luo, Y. Canopy conductance and stand transpiration of Populus simonii Carr in response to soil and atmospheric water deficits in farmland shelterbelt, Northwest China. *Agrofor. Syst.* **91**, 1165–1180 (2017).

Gao, J., Meng, P., Zhang, J., Jia, C. & Ren, Y. Analysis on Transpiration and Difference between Evapotranspiration and Precipitation of Apricot Trees in the Rocky Mountain Area of Northern China. *J. Agrometeorol.* **30**, 538–542 (2009).

Gao, X., Mei, X., Gu, F., Hao, W., Gong, D. & Li, H. Evapotranspiration partitioning and energy budget in a rainfed spring maize field on the Loess Plateau, China. *CATENA* **166**, 249–259 (2018).

Gao, Y., He, H., Zhang, L., Lu, Q., Yu, G. & Zhang, Z. Spatio-temporal variation characteristics of surface net radiation in China
over the past 50 years. *Int. J Geogr. Inf. Sci*. **15**, 1–10 (2013). (in Chinese)

Good, S. P., Noone, D. & Bowen, G. Hydrologic connectivity constrains partitioning of global terrestrial water fluxes. *Science* **349**, 175–177 (2015).

Gu, C. *et al*. Partitioning evapotranspiration using an optimized satellite-based ET model across biomes. *Agric. For. Meteorol.* **259**, 355–363 (2018).

Gu, S. *et al*. Characterizing evapotranspiration over a meadow ecosystem on the Qinghai-Tibetan Plateau. *J. Geophys. Res.: Atmos.* **113**, D08118 (2008).

Guo, L. *The Variations of Water Use Efficiency and Evapotranspiration over a Plantation in the Southern Part of Hilly Areas of NorthChina* (Chinese Academy of Forestry, 2010) (in Chinese)

Han, S., Huang, L., Wang, Z., Wei, Y., Zhang, X. Ecosystem respiration and its controlling factors in the riparian wetland of Yangtze River. *Acta ecologica sinica* **29** (2009).

Hou, L., Wenninger, J., Shen, J., Zhou, Y., Bao, H. & Liu, H. Assessing crop coefficients for Zea mays in the semi-arid Hailiutu River catchment, northwest China. *Agr. water manage.* **140**, 37–47 (2014).

Huang, X., Hao, Y., Wang, Y., Cui, X., Mo, X. & Zhou, X. Partitioning of evapotranspiration and its relation to carbon dioxide fluxes in Inner Mongolia steppe. *J. arid environ.* **74**, 1616–1623 (2010).

Jasechko, S. *et al*. Terrestrial water fluxes dominated by transpiration. *Nature* **496**, 347–350 (2013)

Ji, X., Zhao, W., Kang, E., Jin, B. & Xu, S. Transpiration from three dominant shrub species in a desert-oasis ecotone of arid regions of Northwestern China. *Hydrol. Process.* **30**, 4841–4854 (2016).

Jiang, X., Kang, S., Li, F., Du, T., Tong, L. & Comas, L. Evapotranspiration partitioning and variation of sap flow in female and male parents of maize for hybrid seed production in arid region. *Agr. Water Manage.* **176**, 132–141 (2016).

Jiao, L., Lu, N., Sun, G., Ward, E. & Fu, B. Biophysical controls on canopy transpiration in a black locust (Robinia pseudoacacia) plantation on the semi-arid Loess Plateau, China. *Ecohydrology* **9**, 1068–1081 (2016).

Li, J. *et al*. Characterizing the evapotranspiration of a degraded grassland in the Sanjiangyuan region of Qinghai province. *Acta Prataculturae Sinica* **21**, 223–233 (2012). (in Chinese)

Li, Z., Zhang, Y., Wang, S., Yuan, G., Yang, Y. & Cao, M. Evapotranspiration of a tropical rain forest in Xishuangbanna, southwest China. *Hydrol. Process.* **24**, 2405–2416 (2010).

Lian, X. *et al*. Partitioning global land evapotranspiration using CMIP5 models constrained by observations. *Nat. Clim. Change* **8**, 640–646 (2018)

Lin, E., Jiang, H. & Chen, Y. Water vapor flux variation and net radiation for a Phyllostachys violascens stand in Taihuyuan. *Journal of Zhejiang A&F University* **30**, 313–318 (2013). (in Chinese)

Liu, H. & Feng, J. Seasonal and interannual variations of evapotranspiration and energy exchange over different land surfaces in a semiarid area of China. *J. Appl. Meteorol. Clim.* **51**, 1875–1888 (2012).

Liu, Q., Zeng, H. & Ma, Z. Carbon sequestration of Pinus elliottii plantation in relation with water consumption in subtropical China. *Acta Ecologica Sinica* **28**, 5322–5330 (2008). (in Chinese)

Liu, R., Li, Y. & Wang, Q. Variations in water and CO2 fluxes over a saline desert in western China. *Hydrol. Process.* **26**, 513–522 (2012).

Liu, S., Xu, Z., Zhu, Z., Jia, Z. & Zhu, M. Measurements of evapotranspiration from eddy-covariance systems and large aperture scintillometers in the Hai River Basin, China. *J. Hydrol.* **487**, 24–38 (2013).

Liu, X. *et al*. Partitioning evapotranspiration in an intact forested watershed in southern China. *Ecohydrology* **8**, 1037–1047 (2015).

Liu, X., Zhang, J., Xie, D., Zhuang, J., Shao, Y. & Zhang, S. Temporal variation for canopy transpiration and its cooling properties in a Quercus acutissima forest of suburban Nanjing. *Journal of Zhejiang A&F University* **32**, 529–536 (2015).

Ma, L., Lu, P., Zhao, P., Rao, X., Cai, X. & Zeng, X. Diurnal, daily, seasonal and annual patterns of sap-flux-scaled transpiration from an Acacia mangium plantation in South China. *Ann. For. Sci.* **65**, 9 (2008).

Mo, K., Chen, L., Zhou, J., Fang, X., Kang, M. & Zhang, Z. Transpiration responses of a poplar plantation to the environmental conditions on a floodplain in Northern China. *Acta Ecologica Sinica* **34**, 5812–5822 (2014). (in Chinese)

Ouyang, S. *et al*. Stand Transpiration Estimates from Recalibrated Parameters for the Granier Equation in a Chinese Fir (Cunninghamia lanceolata) Plantation in Southern China. *Forests* **9**, 162 (2018).

Ouyang, Z., Mei, X., Li, Y. & Guo, J. Measurements of water dissipation and water use efficiency at the canopy level in a peach orchard. *AgrI. Water Manage.* **129**, 80–86 (2013).

Qiu, G., Li, C. & Yan, C. Characteristics of soil evaporation, plant transpiration and water budget of Nitraria dune in the arid Northwest China. *Agr. Forest Meteorol.* **203**, 107–117 (2015).

Ren, Q., Meng, P., Zhang, J., Gao, J. & Li, C. Transpiration Variation of the Poplar Shelterbelts and Its Relation to the Meteorological Factors in the Cropland of North China Plain. *Forest Research* **21**, 797–802 (2008).

Schlesinger, W. H. & Jasechko, S. Transpiration in the global water cycle. *Agric. For. Meteorol.* **189–190**, 115–117 (2014).

Shen, Y., Zhang, Y., Scanlon, B. R., Lei, H., Yang, D. & Yang, F. Energy/water budgets and productivity of the typical croplands irrigated with groundwater and surface water in the North China Plain. *Agr. Forest Meteorol.* **181**, 133–142 (2013).

Song, L., Zhu, J., Li, M., Zhang, J., Zheng, X. & Wang, K. Canopy transpiration of Pinus sylvestris var. mongolica in a sparse wood grassland in the semiarid sandy region of Northeast China. *Agr. Forest Meteorol.* **250**, 192–201 (2018).

Tan, Z. *et al*. Rubber plantations act as water pumps in tropical China. *Geophys. Res. Lett.* **38**, L24406 (2011).

Tan, Z., Zhang, Y., Schaefer, D., Yu, G., Liang, N. & Song, Q. An old-growth subtropical Asian evergreen forest as a large carbon sink. *Atmos. Environ.* **45**, 1548–1554 (2011).

Tian, F., Zhao, C. & Feng, Z. Simulating evapotranspiration of Qinghai spruce (Picea crassifolia) forest in the Qilian Mountains, northwestern China. *J. Arid Environ.* **75**, 648–655 (2011).

Wang, D., Wang, G. & Anagnostou, E. Evaluation of canopy interception schemes in band surface models. *J. Hydrol.* **347**, 308–318 (2007).

Wang, W. *et al*. Characteristics of latent heat flux over Cunninghamia lanceolata plantations in Huitong county. *Journal of Central South University of Forestry & Technology* **31**, 192–197 (2011). (in Chinese)

Wang, Z. Energy balance and water vapor flux of snail control and schistosomiasis prevention forests ecosystem in Yangtze River beach land (Chinese Academy of Forestry, 2008). (in Chinese)

Wang-Erlandsson, L., van der Ent, R. J., Gordon, L. J. & Savenije, H. H. G. Contrasting roles of interception and transpiration in the hydrological cycle – Part 1: Temporal characteristics over land. *Earth Syst. Dynam.* **5**, 441–469 (2014).

Wilske, B. *et al*. Poplar plantation has the potential to alter the water balance in semiarid Inner Mongolia. *J. Environ. Manage.* **90**, 2762–2770 (2009).

Yan, M. *et al*. Sapflow-Based Stand Transpiration in a Semiarid Natural Oak Forest on China’s Loess Plateau. *Forests* **7**, 13 (2016).

Zhang, H., Wei, W., Chen, L. & Wang, L. Effects of terracing on soil water and canopy transpiration of Pinus tabulaeformis in the Loess Plateau of China. *Ecol. Eng.* **102**, 557–564 (2017).

Zhang, H., Wei, W., Chen, L. & Yang, L. Evaluating canopy transpiration and water use of two typical planted tree species in the dryland Loess Plateau of China. *Ecohydrology* **10**, 10 (2017).

Zhang, J., Guan, J., Shi, W., Yamanaka, N. & Du, S. Interannual variation in stand transpiration estimated by sap flow measurement in a semi-arid black locust plantation, Loess Plateau, China. *Ecohydrology* **8**, 137–147 (2015).

Zhang, Y. *Energy and water budget of a poplar plantation in suburban Beijing* (Beijing Forestry University, 2010). (in Chinese)

Zhang, Y. *et al*. Multi-decadal trends in global terrestrial evapotranspiration and its components. *Sci. Rep.* **6**, 19124 (2016).

Zhang, Y., Kang, S., Ward, E. J., Ding, R., Zhang, X. & Zheng, R. Evapotranspiration components determined by sap flow and microlysimetry techniques of a vineyard in northwest China: Dynamics and influential factors. *Agr. Water Manage.* **98**, 1207–1214 (2011).

Zhang, Y., Shen, Y., Xu, X., Sun, H., Li, F. & Wang, Q. Characteristics of the water–energy–carbon fluxes of irrigated pear (Pyrus bretschneideri Rehd) orchards in the North China Plain. *Agri. Water Manage.* **128**, 140–148 (2013).

Zhao, P., Kang, S., Li, S., Ding, R., Tong, L. & Du, T. Seasonal variations in vineyard ET partitioning and dual crop coefficients correlate with canopy development and surface soil moisture. *Agr. Water Manage.* **197**, 19–33 (2018).

Zhao, W. *et al*. Evapotranspiration partitioning, stomatal conductance, and components of the water balance: A special case of a desert ecosystem in China. *J. Hydrol.* **538**, 374–386 (2016).

Zhao, W., Chang, X. & Zhang, Z. Transpiration of a Linze jujube orchard in an arid region of China. *Hydrol. Process.* **23**, 1461–1470 (2009).

Zheng, H. *et al*. Spatial variation in annual actual evapotranspiration of terrestrial ecosystems in China: Results from eddy covariance measurements. *J. Geogr. Sci.* **26**, 1391–1411 (2016).

Zhou, G. *et al*. Measured sap flow and estimated evapotranspiration of tropical Eucalyptus urophylla plantations in south China. *Acta Botanica Sinica* **46**, 202–210 (2004).

Zhou, G., Wang, Y. Dynamics of carbon budgets in typical corn and rice ecosystems in Liaohe delta. In: Proceedings of Low Carbon Agriculture Symposium. 133–142 (2010). (in Chinese)

Zhou, S. *et al*. Evapotranspiration of a drip-irrigated, film-mulched cotton field in northern Xinjiang, China. *Hydrol. Process.* **26**, 1169–1178 (2012).

Zhou, S., Liu, W. & Lin, W. The ratio of transpiration to evapotranspiration in a rainfed maize field on the Loess Plateau of China. *Water Sci. Tech.: W. Sup.* **17**, 221–228 (2017).

Zhu, G. *et al*. Energy flux partitioning and evapotranspiration in a sub-alpine spruce forest ecosystem. *Hydrol. Process.* **28**, 5093–5104 (2014).
